# Immune correlates of cardiovascular co-morbidity in HIV infected participants from South India

**DOI:** 10.1186/s12865-022-00498-0

**Published:** 2022-05-17

**Authors:** Bagavathi Kausalya, Shanmugam Saravanan, Suresh Pallikkuth, Rajendra Pahwa, Shelly Rani Saini, Syed Iqbal, Sunil Solomon, Kailapuri G. Murugavel, Selvamuthu Poongulali, Nagalingeswaran Kumarasamy, Savita Pahwa

**Affiliations:** 1 YRG Centre for AIDS Research and Education (YRG CARE), Chennai, India; 2 University of Miami Miller School of Medicine, 1580 NW 10th Avenue; BCRI 712, Miami, FL 33136 USA; 3 Johns Hopkins University School of Medicine, Baltimore, MD USA; 4 VHS-Infectious Diseases Medical Centre, Chennai, India

**Keywords:** HIV and comorbidities with cardiovascular diseases, Inflammation and CVD in HIV, CD4 count and HIV cardiovascular disease, ART and CVD risk, Microbial translocation, CVD in HIV

## Abstract

**Background:**

Understanding the immune correlates of cardiovascular disease (CVD) risk in HIV infection is an important area of investigation in the current era of aging with HIV infection. Less is known about CVD risk and HIV infection in developing nations where additional risk factors may be playing a role in the CVD development. In this study, we assessed the effects of systemic inflammation, microbial translocation (MT), T cell immune activation (IA), and nadir CD4 counts on cardiac function and arterial stiffness as markers of subclinical atherosclerosis in HIV-infected individuals.

**Methods:**

People with HIV (PWH) who were ART naïve (n = 102) or virally suppressed on ART (n = 172) were stratified on nadir CD4 counts and compared to HIV-uninfected controls (n = 64). Determination was made of cardiac function via radial pulse wave and carotid intima thickness (C-IMT) measurements. Plasma biomarkers of inflammation and MT by ELISA or multiplex assays, and immune activation (IA) of T cells based HLA-DR and CD38 expression were investigated by flow cytometry. T-test, Mann–Whitney U test, and Spearman correlation were used to analyze study parameters.

**Results:**

Reduction in cardiac function with lower cardiac ejection time (*p* < 0.001), stroke volume (*p* < 0.001), cardiac output (*p* = 0.007), higher arterial stiffness (*p* < 0.05) were identified in ART-naïve participants, compared to PWH on ART (*p* < 0.05). No significant difference in C-IMT values were noted. Higher inflammatory and MT markers were found in the ART-naïve group compared to treated group who were comparable to uninfected participants, except for having higher TNF-α (*p* < 0.001) and sCD14 (*p* < 0.001). Immune activation of CD4 and CD8 T-cells was greater in ART-naïve participants compared to ART-treated and uninfected controls (*p* < 0.05). Lower nadir CD4 counts, higher inflammation, and higher MT predicted poor cardiac measures in the ART-naïve with nadir CD4 < 200cells/mm^3^ manifesting the highest arterial stiffness, and lowest cardiac function, whereas ART-treated, even with nadir < 200 cells/mm^3^ were similar to uninfected in these measures.

**Conclusions:**

In HIV-infected individuals, initiation of ART even at nadir of < 200 cells/mm^3^ may prevent or reverse cardiovascular disease outcomes that are easily measurable in low income countries.

**Supplementary Information:**

The online version contains supplementary material available at 10.1186/s12865-022-00498-0.

## Background

Understanding risk and mechanism of Cardiovascular disease (CVD) in HIV infection is under intense investigation in the developed world, but less is known about this comorbidity in developing nations where additional risk factors may be playing a role [1–3]. The present study was focused on people with HIV (PWH) from the Indian subcontinent who in general are more predisposed to CVD, with its occurrence at least a decade earlier in the general population than in people of European ancestry [4, 5]. Risk of mortality due to CVD before the age of 70 years is 52% among Indians as compared to 23% in Western populations [6]. The impact of HIV infection on CVD in people from India is understudied. Overall, the risk of CVD in PWH on ART has been found to be twice that of HIV-negative persons [7] but other factors can further alter these dynamics. This issue is particularly important in the “treat all” era where the risk for comorbidities persist despite virus suppression with antiretroviral therapy (ART) [8–10] and decline in AIDS-related mortality [11, 12]. However in developing nations, patients often do not enter into care until late in the disease, and the drugs used in ART regimens may vary and thereby influence patient outcomes [13–16]. The strategies for management of antiretroviral therapy (SMART) study that included people of various backgrounds clearly established the importance of continuous ART in reducing risk of CVD compared to intermittent ART [17], but the veterans aging cohort (VAC) Study using data collected only on veterans found that ART was associated with an increased risk of acute MI [18]. As our understanding of the potential impact of HIV infection on CVD evolves, more data are needed to delineate the detrimental effects of HIV on cardiac function in culturally and ethnically diverse populations from various parts of the world including low resource countries.

Among pathogenic factors that influence CVD the role of immune activation and inflammation in HIV-associated atherosclerosis has received much attention. A majority of published studies have focused on the role of the innate immune system, such as markers of monocyte and macrophage activation [19–21] on HIV-associated atherosclerosis. Increased inflammatory response has been implicated in CVD for the general population and may be partly driving the association between HIV and CVD, contributing to the persistent risk noted after adjusting for traditional CVD risk factors [15, 22–24]. For example, the inflammatory cytokine IL-6 was found to be elevated in progressive HIV infection [25–29] and to predict mortality in HIV infected participants [30, 31]. In addition, elevations in soluble inflammatory receptors, such as Tumor Necrosis Factor Receptor (TNFR) I and II, have been implicated in myocardial dysfunction after acute coronary syndromes, as well as in recurrent myocardial infarction (MI) and cardiac death [32, 33]. An association of gut-associated microbial translocation (MT) based on soluble CD14 (sCD14) and plasma lipopolysaccharide (LPS) levels and CVD risk has been reported in PWH [34].

Arterial elasticity along with carotid-intima media thickness (C-IMT) measurements are valuable tools to identify early vascular functional and structural abnormalities. These measures are easily adapted to outpatient facilities and have provided valuable information in studies of the influence of ART regimens on traditional CVD risk factors [34–37]. Torriani et al.reported improved brachial flow measures within 4 weeks of initial ART use, which was associated with a decline in HIV RNA levels [38]. Delayed entry into care can lead to initiation of ART at lower nadir CD4 counts, which have been associated with higher C-IMT [39, 40] and MI events [41] but this is not well established and controversies exist [40, 42]. Adopting measures such as C-IMT in outpatient facilities in LRC can potentially facilitate implementation of CVD prevention strategies in PWH [39, 43–46]. Data are needed to fill in the gaps in understanding how C-IMT and arterial stiffness are affected by nadir CD4 counts as well as the role of gut-associated MT. The present study analyzed the functional and structural changes of arterial vasculature using practical tools in an outpatient setting in treatment-naïve and ART-experienced HIV + participants from South India across different CD4 nadirs and their associations with T-cell immune activation, inflammation, and microbial translocation (MT). Our findings show that adverse impact of HIV on these measures of cardiac function in patients with low CD4 nadirs can potentially be reversed with ART initiation.

## Methods

### Study setting and subjects

Recruitment of participants for this study was conducted during 2014–2016 at YRG CARE, a tertiary care center in Chennai located in South India providing patient care to more than 20,000 PWH. The study enrolled 274 male and female participants with chronic HIV infection (HIV +) and 64 HIV-uninfected healthy controls (HC) at age > 18 yr. Among the HIV + participants, 102, with 50 males and 52 females were ART-naïve with no pre-exposure to any ART (Group 1). 172 with 114 males and 58 females were on ART for > 12 months viral suppression with plasma viral load of < 40 copies/mL on two consecutive measurements and CD4 counts > 500/μL (Group 2). HC were categorized as Group 3 with 18 males and 46 females. All the HC participants were from the same socio-demographic background as the HIV + participants. HC on pre-exposure prophylaxis, and pregnant women were excluded. In Groups 1 and 2, HIV + participants were stratified based on nadir CD4 counts of < 200 cells/µL (groups 1a and 2a); 200–350 cells/µL (groups 1b and 2b) and > 350 cells/µL (groups 1c and 2c). The study was approved by both YRG CARE and University of Miami institutional review boards, with written informed consent obtained from all enrolled participants. A detailed interview was conducted at time of enrollment to collect demographic information along with screening tests for HBV and HCV co-infections and cardiovascular assessment including a complete lipid profile. All measures of cardia function were obtained in the outpatient setting coupled to a clinic visit.

### Sample processing and storage

40 ml venous blood was collected in EDTA containing sterile vacutainer tubes (BD Biosciences) at the visit. Plasma collection was performed by centrifuging the blood at 400 g for 10 min and plasma was collected and further centrifuged at 1000 g for 15 min to remove the platelets. Plasma was stored at −80 °C until further use. Peripheral blood mononuclear cells (PBMC) were collected from the whole blood by density gradient method and cells were cryopreserved in liquid N2. All the laboratory procedures used sterile endotoxin free tubes.

### Carotid Intima-media Thickness (C-IMT) measurement

Doppler study of carotid and vertebral arteries was performed by B-mode ultrasound for all participants. Thickness of intima-media complex was measured in both the right and left common carotid arteries.

### Measurement of cardiac functions and arterial stiffness

Stiffness in the arterial system was estimated by pulse-wave velocity (PWV) using the HDI/PulseWave CR-2000 (Hypertension Diagnostics, Inc., Eagan, MN), a diagnostic tool that was previously applied in the International Network for Strategic Initiatives in Global HIV Trials (INSIGHT) Strategic Timing of Anti Retroviral Treatment (START) arterial stiffness sub-study [47]. Along with Large Artery Elasticity index (LAE) and Small Artery Elasticity (SAE) index measures, systemic vascular resistance (SVR) and total vascular impedance (TVI) were measured as arterial stiffness parameters with measurements of pulse rate, stroke volume, stroke volume index, cardiac output, cardiac index, and cardiac ejection time to ascertain cardiac functioning.

### Quantification of inflammatory and MT markers

The plasma inflammatory and MT markers TNFR-I, TNFR-II and sCD14 were measured using RayBio Tech ELISA kits, (RayBiotech, Inc., GA) after appropriate dilution of the plasma according to manufacturer’s protocol. Cytokines IL-1b, IL-2, IL-6, IL-8, IL-10, IL-12 (p70), IL-17, IFN-α2, IFN-γ and TNF-α levels were determined in undiluted plasma using Milliplex cytokine magnetic bead panel in the Magpix instrument (Luminex Corporation). These markers were selected based on their known association with systemic inflammation and immune activation. Median fluorescent intensities (MFI) were analyzed and cytokine levels were expressed as pg/mL. LPS was measured in plasma samples using the Limulus amebocyte lysate chromogenic endpoint assay (Lonza, MD, USA).

### Analysis of markers of immune activation and exhaustion

Thawed cryopreserved PBMC were rested overnight. After overnight rest, cell recovery was between 75 and 85% with a viability of > 90% for all the samples. 1 × 10^6^ cells were stained with antibodies against different cell surface markers in the dark at room temperature along with live-dead discriminator (Aqua). Cells were acquired on a BD LSRFortessa (BD Bioscience, San Jose, CA). Data were analyzed using FlowJo software (TreeStar V10.02, Ashland, OR). Frequencies of desired subsets were determined in gated live (Aqua^–^) cell populations. Total CD4 + and CD8 + T-cells were analyzed for immune activation based on the co-expression of HLA-DR and CD38  [48].

### Statistical analysis

**Fig. 1:**
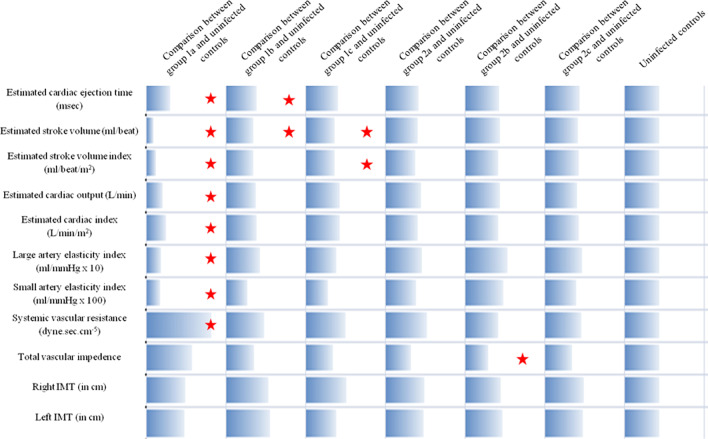
Comparison of cardiac functions and arterial stiffness parameters between the study groups with the uninfected control group: All the parameters were converted to fold changes with uninfected controls, while fold change for uninfected controls was taken as ‘1’. *Indicates statistical significance with *p* < 0.05

Descriptive statistics such as percentages, means and standard deviation, and median and interquartile ranges were used to describe the demographic characteristics of the study population. Levene’s statistic was used to test for homogeneity of variances followed by independent samples T-test, Mann–Whitney U-test, ANOVA and Welch ANOVA if homogeneity of variances was not attained. Tukey or Games Howell post-hoc tests were used to compare various quantitative variables between the groups. Fold change over uninfected controls was calculated by dividing the HIV infected group data by uninfected controls data. In Fig. 1, uninfected controls are plotted as ‘1’ and values above and below 1 indicate fold change that is greater and lower respectively. Spearman correlation was used to identify associations of cardiac functional and structural parameters with inflammatory indices, microbial translocation, T-cell activation markers, and CD4 counts.

## Results

### Demographic characteristics of study population

Age was matched for all groups in the study. At the time of enrollment, 140 participants on ART were on first-line reverse transcriptase inhibitors (RTI; AZT/D4T/TDF + 3TC/FTC + EFV/NVP), while 32 were on PI-based (RTV-boosted LPV) second-line therapy. When stratified based on smoking habits, 8% of Group 1, 13% of Group 2 and 8% of the Group 3 were found to be current smokers, 24.6% of Group 1, 30% of Group 2 and 21% of the Group 3 were found to be past smokers, and 67.5% of Group 1 and 57% of Group 2 were found to be never smokers. Of the 274 HIV-infected participants, two were found to be HBV and HCV-positive. Characteristics of the study groups are shown in Table 1.

**Table 1:** Characteristics of the study cohorts

Demographic characteristics	Group 1 (n = 102)	Group 2 (n = 172)	Group 3 (n = 64)	*p-*value Group 1 vs 2	*p-*value Group 1 vs 3	*p-*value Group 2 vs 3	Group 1a (n = 29)	Group 1b (n = 22)	Group 1c (n = 51)	*p-*value Group 1a vs 1b	*p-*value Group 1a vs 1c	*p-*value Group 1b vs 1c	Group 2a (n = 51)	Group 2b (n = 51)	Group 2c (n = 70)	*p-*value Group 2a vs 2b	*p-*value Group 2a vs 2c	*p-*value Group 2b vs 2c
Age (in years)	36.5 ± 5.8	38.6 ± 5.9	36.8 ± 7.09	0.068	0.795	0.07	37.90 ± 6.28	36.82 ± 6.09	35.71 ± 5.41	0.542	0.105	0.441	38.82 ± 5.48	38.10 ± 6.17	38.94 ± 6.17	0.532	0.913	0.459
Gender (M/F)	50/52	114/58	18/46				17/12	15/7	18/33				35/16	35/16	44/26			
Known period of infection (in months)	15 (2, 42)	72 (39.5, 92)	–	** < 0.001**	–	–	2 (1, 19)	2 (1, 3)	35 (16.5, 49)	0.418	**0.001**	** < 0.001**	74 (31, 114.5)	69 (41.5, 90.5)	72 (44, 81)	0.416	0.326	0.929
Nadir CD4 + T-cell count (cells/ mm^3^)	353 (167, 544)	307 (186.5, 427)	–	0.118	–	–	70.5 (45.5, 119)	281 (238, 314)	544 (454, 631.5)	** < 0.001**	** < 0.001**	** < 0.001**	119 (83, 165.5)	277 (227.5, 313)	450 (392, 540)	** < 0.001**	** < 0.001**	** < 0.001**
CD4 + T-cell count at the time of study entry (cells/ mm^3^)	352 (167, 581)	722 (560, 935)	–	** < 0.001**	–	–	70.5 (45.5, 119)	281 (238, 314)	581 (495, 771.5)	< **0.001**	** < 0.001**	** < 0.001**	553 (422.5, 731.5)	659 (560, 817.5)	871 (709, 1044)	**0.038**	** < 0.001**	** < 0.001**
Duration on ART (in months)	–	43 (22, 72)	–	–	–	–	–	–	–				52 (25.5, 85)	32 (19, 50.5)	53 (22, 74)	**0.021**	0.248	0.068
Body Mass Index (Kg/m^2^)	22.7 (20.1, 26.3)	22 (19, 25.8)	24.6 (22.5, 27.1)	0.844	**0.013**	**0.006**	21.4 (16.85, 23)	22.3 (20.1, 27.2)	24.2 (21.7, 27.3)	0.072	** < 0.001**	0.269	24.4 (19.9, 27.8)	23 (19.1, 25.85)	22.4 (19.2, 25.3)	0.133	**0.02**	0.392

Group 1, ART naïve patients; Group 2, Patients on ART; Group 3, HIV-uninfected controls. Groups 1a and 2a, nadir CD4 + T-cell count < 200 cells/mm^3^; Groups 1b and 2b, nadir CD4 + T-cell count 200–350 cells/mm^3^; Groups 1c and 2c, On ART patients with nadir CD4 + T-cell count > 350 cells/mm^3^. All descriptive variables are provided as median and interquartile ranges except for age, which is provided as mean ± SD. Independent samples T-test was used to calculate *p*-value between the groups for ‘age’ which was normally distributed, while Mann–Whitney U-test was used for other non-normally distributed variables. Statistically significant values are in bold

### Cardiac function parameters are altered in treatment naïve participants

HIV infection may increase the risk of CVD, with previous studies showing increased risk of subclinical atherosclerosis [49]. The data in Table 2 shows that Group 1 had significantly lower cardiac ejection time (*p* < 0.001), stroke volume (*p* < 0.001), and cardiac output (*p* = 0.008) than Group 2. Group 3 had superior cardiac function compared to Group 1 in all of the same cardiac function parameters. Groups 2 and 3, however, did not vary significantly in cardiac function except for a significantly lower vascular impedance in group 2 (Table 2). Thus, ART treatment was found to potentially be cardioprotective, with CVD risk factors in Group 2 being comparable to healthy participants. Low CD4 counts have shown variable results in terms of CVD risk [50]. Among the subgroups, group 1a had the lowest cardiac functions of all three subgroups (Additional File 1: Table S1). Compared to uninfected controls, the fold change in cardiac measures were significantly lower in the group 1a (Fig. 1). Their cardiac ejection time, stroke volume, stroke volume index, cardiac output and cardiac index were significantly lower than groups 1b and 1c (Additional File 1:Table S1). Fold changes in cardiac function parameters in group 1c were comparable to uninfected controls with the exception of a lower stroke volume and stroke volume index (Fig. 1). Interestingly, Group 2a who had initiated ART at CD4 nadirs < 200 cells/mm^3^ showed minimal differences in Cardiac function measures from Group 3. These results show that PWH on ART who achieve CD4 reconstitution are near normal to HC in cardiac measures employed in this study, but may show some residual abnormalities e.g. in vascular impedance amongst patients who started ART at a low nadir CD4 count of < 200 cells/mm^3^.

**Table 2:** Measures of cardiac functions, and arterial stiffness in HIV + treatmentnaïve, on ART, and HIV uninfected participants

Clinical parameters	Group 1; (n = 102)	Group 2; (n = 172)	Group 3; (n = 64)	*p-*value; Gp 1 vs 2	*p-* value; Gp 1 vs 3	*p-* value; Gp 2 vs 3
Estimated cardiac ejection time (millisec)	288 (264, 305)	305 (280, 325)	304 (288, 324.5)	< 0.001*	< 0.001*	0.623
Estimated stroke volume (ml/beat)	61 (50, 72)	71 (58, 80)	70 (60.5, 80)	< 0.001*	< 0.001*	0.661
Estimated stroke volume index (ml/beat/m^2^)	38 (32, 44)	42 (36, 49)	43 (38.5, 48)	< 0.001*	< 0.001*	0.434
Estimated cardiac output (L/min)	4.8 (4.3, 5.3)	5 (4.5, 5.6)	5.1 (4.7, 5.65)	0.008*	0.005*	0.383
Estimated cardiac index (L/min/m^2^)	3 (2.7, 3.3)	3.05 (2.8, 3.3)	3.1 (2.95, 3.4)	0.077	0.004*	0.071
Large artery elasticity index						
(ml/mmHg × 10)	12.8 (9.7, 16.2)	14.5 (11.5, 18)	13.1 (10.25, 16)	0.001*	0.356	0.06
Small artery elasticity index						
(ml/mmHg × 100)	4.4 (2.8, 5.9)	4.75 (3.4, 6.5)	5.4 (3.55, 6.75)	0.019*	0.01*	0.526
Systemic vascular resistance						
(dyne.sec.cm^−5^)	1431 (1256, 1663)	1325.5 (1162, 1528)	1315 (1178.5, 1542)	0.003*	0.006*	0.735
Total vascular impedance (dyne.sec.cm^−5^)	127 (102, 155)	119.5 (99, 140)	128 (113, 155)	0.046*	0.64	0.024*
Right IMT (in cm)	0.05 (0.05, 0.06)	0.05 (0.04, 0.06)	0.05 (0.04, 0.06)	0.172	0.036*	0.269
Left IMT (in cm)	0.05 (0.04, 0.06)	0.05 (0.04, 0.06)	0.05 (0.04, 0.06)	0.212	0.667	0.142

Group 1; Naïve patients, Group 2; Patients on ART, Group 3; HIV-uninfected controls. All continuous variables are presented as median and interquartile ranges in parenthesis. Mann–Whitney U-test was used to calculate *p*-value between the study groups

*Indicates statistically significant differences

ART = anti-retroviral therapy, IMT = intima-media thickness, ml = milliliter, L = Liter

### Higher arterial stiffness and vascular resistance in treatment naïve participants

Progression of HIV infection and declining CD4 counts have shown to increase arterial stiffness [51]. We observed that Group 2 had higher LAE (*p* = 0.001) and SAE (*p* = 0.019) than Group 1 as well as lower SVR (*p* = 0.003) and total vascular impedance (*p* = 0.046), as shown in Table 2. Group 3 did not vary significantly in arterial stiffness parameters from Group 2 while right IMT was different between Group 3 and Group 1 (Table 2). Of all the study populations, treatment-naïve participants had the greatest arterial stiffness and vascular resistance. The results in Additional File 1: Table S1 show that group 1a had increased arterial stiffness based on lower LAE and higher SVR compared to other treatment naïve groups (*p* < 0.05). Participants in group 1c had comparable levels of cardiac functions and arterial elasticity to uninfected controls, while 1a had altered cardiac measures (Fig. 1). Among the virologically suppressed participants in Group 2, those in groups 2a (*p* = 0.021) and 2c (*p* = 0.068) had longer duration of treatment than group 2b (Table 1). From the data in Additional File 1: Table S1, no significant difference in arterial stiffness was noted between treated groups initiating ART in different nadir CD4 groups. Thus, ART may preserve arterial elasticity regardless of starting nadir CD4 counts.

### Higher nadir CD4 counts correlated with better cardiac function

**Fig. 2:**
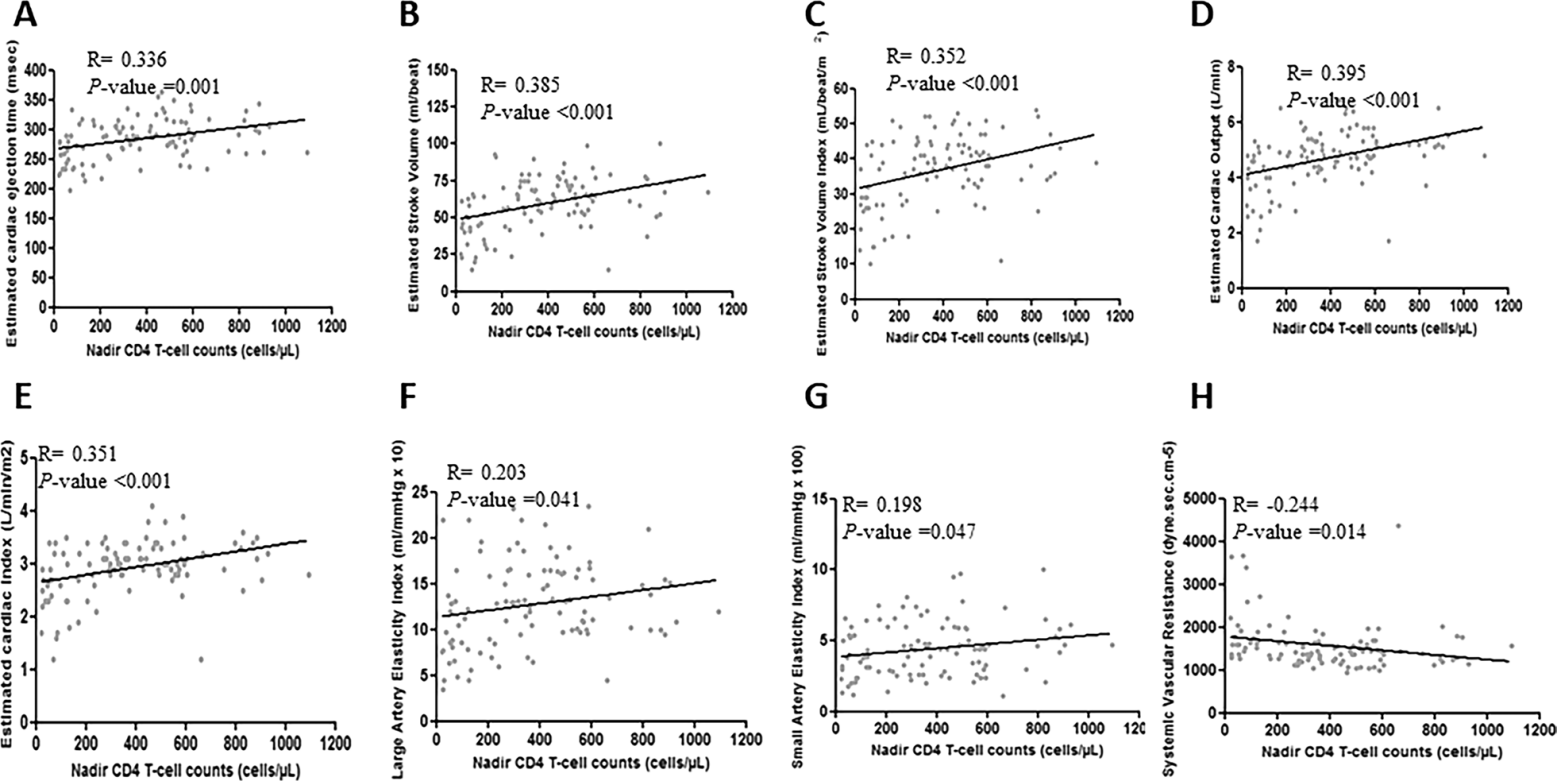
Association of nadir CD4 counts with cardiac functioning and arterial stiffness in naïve participants. **A** Association of nadir CD4 counts with estimated cardiac ejection time. **B** Association of nadir CD4 counts with estimated stroke volume. **C** Association of nadir CD4 counts with estimated stroke volume index. **D** Association of nadir CD4 counts with estimated cardiac output. **E** Association of nadir CD4 counts with estimated cardiac index. **F** Association of nadir CD4 counts with large artery elasticity index. **G** Association of nadir CD4 counts with small artery elasticity index. **H** Association of nadir CD4 counts with systemic vascular resistance

Lower nadir CD4 count is a marker of advanced disease and of more virus-induced CD4 T-cell destruction [52]. In the group 1, higher nadir CD4 counts correlated positively with better cardiac function, including higher cardiac ejection time, higher stroke volume, higher stroke volume index, higher cardiac output, higher cardiac index, LAE, SAE, and lower SVR, (*p* < 0.05; Fig. 2; Table 3). Even though the significance noted was not very strong, the findings suggest that severity of immune compromise in HIV + persons increases the risk for CVD, underscoring the importance of initiating ART as early as possible.

**Table 3:** Correlation of CD4 T-cell counts with sub-clinical CVD markers in the HIV + treatment naïve group

Sub-clinical CVD markers	Statistics	CD4 T-cell counts at the time of study enrollment
Estimated cardiac ejection time (msec)	Spearman (R)	0.336*
	*p* value	0.001
Estimated stroke volume (ml/beat)	Spearman (R)	0.385*
	*p* value	< 0.001
Estimated stroke volume index (ml/beat/m^2^)	Spearman (R)	0.352*
	*p* value	< 0.001
Estimated cardiac output (L/min)	Spearman (R)	0.395*
	*p* value	< 0.001
Estimated cardiac index (L/min/m^2^)	Spearman (R)	0.351*
	*p* value	< 0.001
Large artery elasticity index (ml/mmHg × 10)	Spearman (R)	0.203*
	*p* value	0.041
Small artery elasticity index (ml/mmHg × 100)	Spearman (R)	0.198*
	*p* value	0.047
Systemic vascular resistance (dyne.sec.cm^−5^)	Spearman (R)	−0.244*
	*p* value	0.014

*Indicates statistical significance. Spearman correlation was used to identify the correlations

### T-cell immune activation markers in treatment naïve and ART groups associated with poor cardiac outcomes

**Fig. 3:**
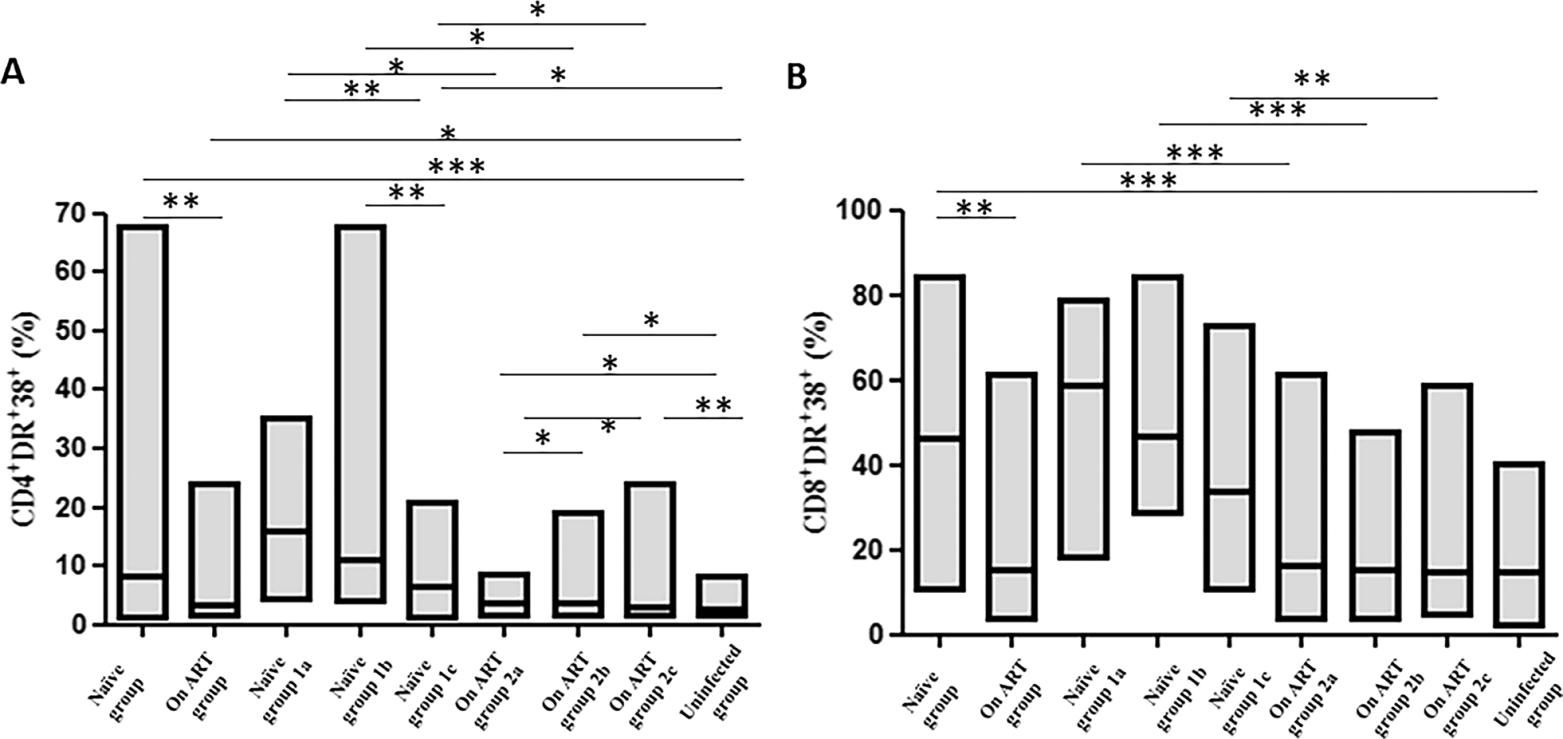
CD4 and CD8 T-cell immune activation between treatment naïve and ART-treated participants stratified by nadir CD4 T-cell counts in comparison to the uninfected control group. CD4 and CD8 T cell immune activation were analyzed by Flow cytometry based on the surface expression of HLA-DR and CD38 dual positive CD4 and CD8 T cells. **A**, DR^+^38^+^: CD4 T-cell activation; **B**, CD8^+^DR^+^38^+^: CD8 T-cell activation; group 1a: Untreated participants   with nadir CD4 T-cell counts < 200 cells/mm^3^; group 1b: Untreated participants  with nadir CD4 T-cell counts 200–350 cells/mm^3^; group 1c: Untreated participants with nadir CD4 T-cell counts > 350 cells/mm^3^; group 2a: On ART participants with nadir CD4 T-cell counts < 200 cells/mm^3^; group 2b: On ART participants with nadir CD4 T-cell counts 200–350 cells/mm^3^; group 2c: On ART patients with nadir CD4 T-cell counts > 350 cells/mm^3^. Lines with asterix (*) show the significant difference (*p* < 0.05) between indicated groups with *p < 0.05, **p < 0.01, and ***p < 0.001

Chronic immune activation and inflammation during HIV infection are thought to increase CVD progression [53, 54]. Our results revealed that CD4 and CD8 T-cell immune activation measured by co-expression of HLA-DR and CD38  was higher in Group 1 compared to Group 2 (*p* < 0.001) and Group 3 (*p* < 0.001; Table S2; Fig. 3). In the context of nadir CD4, groups 1a & 1b had higher immune activation in both CD4 and CD8 T-cells (*p* < 0.05) than group 1c. Group 1c also showed higher T-cell immune activation (*p* < 0.001) compared to group 3. Among group 2, CD4 T-cell immune activation was lowest in the group with the highest CD4 nadir (group 2c) compared to 2a (*p* = 0.001) and 2b (*p* = 0.03) and the levels were still higher than uninfected controls (Fig. 3a). CD8 T-cell immune activation did not differ between different nadir CD4 in group 2 (Fig. 3b). Comparing respective CD4 nadirs in groups 1 and 2, CD4 and CD8 T-cell immune activation was lower in group 2a, b, c than in groups 1a, b, c respectively (*p* < 0.01). In group 1, an inverse correlation was noted between CD4 and CD8 T-cell immune activation and cardiac ejection time, stroke volume, cardiac output, and cardiac index (*p* < 0.05; Table 4). In group 2 as well CD8 T-cell immune activation was inversely correlated with large and small artery elasticity (*p* < 0.05; Table 4). These results imply that lowering of both CD4 and CD8 T-cell  immune activation by ART may be cardioprotective.

**Table 4:** Correlation coefficient of cardiac functional and arterial stiffness markers with T-cell immune activation in HIV + treatment naïve and on ART participants

Cardiac functional and arterial stiffness markers	Study groups	CD4 T−cell activation (CD4^+^ HLA−DR^+^ 38^+^)	CD8 T−cell activation (CD8^+^ HLA−DR^+^ 38^+^)
Estimated cardiac ejection time (msec)	Group 1	−0.341*	−0.212*
	Group 2	−0.036	−0.121
Estimated stroke volume (ml/beat)	Group 1	−0.378*	−0.241*
	Group 2	−0.048	−0.177*
Estimated stroke volume index (ml/beat/m^2^)	Group 1	−0.358*	−0.184
	Group 2	0.07	−0.117
Estimated cardiac output (L/min)	Group 1	−0.369*	−0.308*
	Group 2	−0.002	−0.089
Estimated cardiac index (L/min/m^2^)	Group 1	−0.328*	−0.226*
	Group 2	0.078	−0.009
Large artery elasticity index (ml/mmHg × 10)	Group 1	−0.192	−0.06
	Group 2	−0.069	−0.163*
Small artery elasticity index (ml/mmHg × 100)	Group 1	−0.076	−0.159
	Group 2	−0.017	−0.171*
Systemic vascular resistance (dyne.sec.cm^−5^)	Group 1	0.151	0.12
	Group 2	0.006	0.136
Total vascular impedance (dyne.sec.cm^−5^)	Group 1	0.12	0.086
	Group 2	0.025	0.113

Group 1; treatment Naïve participants , Group 2; Partcipants on ART

*Indicates statistically significant correlations. Spearman correlation was used to identify correlations

### Plasma inflammatory markers in naïve and ART groups and their association with sub-clinical CVD progression

Various markers of inflammation have been associated with CVD and increased mortality during HIV infection [55–59]. We found that Group 1 had higher amounts of certain plasma inflammatory cytokines compared to Groups 2 (*p* < 0.05) and 3 (*p* < 0.05), including IFNα2, IL-10, IL-6, TNFα, TNFR-1, and TNFR-2 (Table S2). Groups 2 and 3 had similar plasma inflammatory cytokines with the exceptions of TNF, and IL-12 which were higher in Group 2 (*p* = 0.001). Not only did Group 1 have the greatest T-cell immune activation, but also the highest amounts of inflammatory cytokines. Elevated IL-6 levels in naïve participants were associated with altered cardiac measures, including lower stroke volume and stroke volume index, lower cardiac output, lower LAE, and higher SVR (*p* < 0.05; Table 5). Higher TNFR1 and TNFR2 were also associated with poor cardiac measures parameters in group 1 (*p* < 0.05; Table 5). In the absence of ART, elevated markers of inflammation appear to have a detrimental role on CVD progression.

**Table 5:** Correlation coefficient of cardiac functional and arterial stiffness markers of inflammation and microbial translocation in HIV + treatment naïve participants

Sub-clinical CVD markers	Statistics	TNFR1 (pg/mL)	TNFR2 (pg/mL)	IFN-α2 (pg/mL)	IFN-γ (pg/mL)	IL10 (pg/mL)	IL2 (pg/mL)	IL6 (pg/mL)	TNF-α (pg/mL)	LPS (pg/mL)	sCD14 (ng/mL)
Estimated cardiac ejection time (msec)	Spearman (R)	−0.223*	−0.154	0.165	0.109	0.136	0.183	−0.071	−0.101	−0.186	−0.261*
	*p* value	0.025	0.125	0.099	0.277	0.175	0.068	0.48	0.315	0.063	0.008
Estimated stroke volume (ml/beat)	Spearman (R)	−0.370*	−0.324*	0.082	−0.012	0.036	0.096	−0.257*	−0.222*	−0.259*	−0.362*
	*p* value	< 0.001	0.001	0.414	0.908	0.724	0.341	0.009	0.026	0.009	< 0.001
Estimated stroke volume index (ml/beat/m^2^)	Spearman (R)	−0.350*	−0.264*	0.142	0.049	0.096	0.155	−0.221*	−0.195	−0.323*	−0.338*
	*p* value	< 0.001	0.008	0.156	0.627	0.339	0.123	0.027	0.05	0.001	0.001
Estimated cardiac output (L/min)	Spearman (R)	−0.377*	−0.290*	0.057	0.003	0.043	0.08	−0.251*	−0.203*	−0.219*	−0.263*
	*p* value	< 0.001	0.003	0.57	0.978	0.672	0.429	0.012	0.042	0.028	0.008
Estimated cardiac index (L/min/m^2^)	Spearman (R)	−0.329*	−0.171	0.134	0.095	0.134	0.158	−0.183	−0.164	−0.292*	−0.209*
	*p* value	0.001	0.087	0.18	0.347	0.181	0.115	0.067	0.102	0.003	0.036
Large artery elasticity index (ml/mmHg × 10)	Spearman (R)	−0.310*	−0.302*	0.05	−0.146	0.015	−0.036	−0.316*	−0.131	−0.151	−0.273*
	*p* value	0.002	0.002	0.621	0.146	0.884	0.72	0.001	0.193	0.133	0.006
Small artery elasticity index (ml/mmHg × 100)	Spearman (R)	−0.098	−0.126	0.213*	0.124	0.069	0.234*	−0.069	0.009	−0.239*	−0.075
	*p* value	0.332	0.209	0.033	0.217	0.494	0.019	0.495	0.93	0.016	0.453
Systemic vascular resistance (dyne.sec.cm^−5^)	Spearman (R)	0.355*	0.292*	−0.077	0.005	−0.066	−0.06	0.334*	0.15	0.313*	0.098
	*p* value	< 0.001	0.003	0.442	0.964	0.515	0.553	0.001	0.134	0.001	0.331
Total vascular impedance	Spearman (R)	0.348*	0.323*	−0.042	0.107	−0.025	0.047	0.363*	0.146	0.113	0.165
	*p* value	< 0.001	0.001	0.675	0.285	0.802	0.643	< 0.001	0.144	0.262	0.099

*Indicates statistically significant correlations. Spearman correlation was used to identify correlations

### Plasma gut microbial translocation markers in treatment naïve and ART groups and their association with sub-clinical CVD progression

Bacterial translocation may lead to increased immune activation in HIV infection causing CVD progression [15, 34, 60]. Group 1 had higher plasma LPS compared to Groups 2 (*p* < 0.001) and 3 (*p* < 0.001), while plasma LPS levels in Groups 2 and 3 were not different (Table S2). Group 1 also had higher sCD14 compared to Group 3 (*p* = 0.004) but not to Group 2. In addition, sCD14 was higher in Group 2 compared to Group 3 (*p* < 0.001; Table S2). Our results show that the markers of microbial translocation are highest among treatment-naïve participants, with partial elevations seen even with ART treatment.

Similar to elevated IL-6 levels, elevated LPS levels in group 1 were associated with poor cardiac measures, including lower stroke volume and stroke volume index, lower cardiac output and cardiac index, lower LAE, and higher SVR (*p* < 0.05; Table 5). Likewise, elevated sCD14 was associated with lower cardiac ejection time, lower stroke volume and stroke volume index, lower cardiac output and cardiac index, and lower LAE (*p* < 0.05; Table 5). Overall, Similar to inflammatory biomarkers, microbial translocation was also correlated with poor cardiac function.

## Discussion

We assessed sub-clinical CVD progression and cardiac health in terms of functional and structural cardiac changes in HIV-infected participants from South India. Our study identified decreased cardiac functioning and elevated inflammatory and T-cell immune activation markers in ART-naïve participants; however, it did not identify significant increases in C-IMT measurements during HIV-infection. These results indicate that ART-induced viral suppression may have cardio-protective effects, as seen with superior cardiac function and reduced arterial stiffness in ART-treated participants. In addition, lower nadir CD4 count was identified as a major factor associated with poor cardiac function in treatment-naïve participants.

The effects of HIV infection on the risk of CVD have shown conflicting data, with some studies showing increased risk of subclinical atherosclerosis [49], and others finding no association between HIV infection and CVD risk [18, 61, 62]. Many studies in people living with HIV have shown an effect of low CD4 + T-cell count on MI risk [63, 64], carotid atherosclerosis [40, 65], arterial stiffness [51], and endothelial dysfunction [21, 50, 66–68]. Our results demonstrated that low nadir CD4 counts were associated with reduced cardiac function, especially in ART-naïve participants, compared to those with nadir CD4 counts > 350cells/mm^3^. In addition, participants who initiated ART and had achieved viral suppression had better cardiac function parameters. The initiation of ART in HIV + participants was associated with reduced risk of CVD compared to naïve participants [18, 69]. Thus, our data highlights the importance of higher CD4 counts and early ART initiation as people who started ART at higher nadir CD4 counts were associated with decreased CVD risk in both naïve and ART-treated groups.

HIV infection has been shown to increase the risk of coronary artery disease through increasing plaque formation and arterial stiffness [45, 46, 49, 70, 71]. In our group 1, C-IMT levels did not vary with differences in nadir CD4; however, reduced LAE and SAE and elevated SVR and vascular impedance were noted with treatment naïve low nadir CD4 group. LAE and SAE as measures of arterial stiffness are effective markers of CVD, with central measures being able to independently predict future clinical events [72–74]. Changes in arterial stiffness was found to precede the development of atherosclerosis, which could explain why early changes in C-IMT were not found [75]. Other traditional risk factors, such as smoking, also contribute to atherosclerotic disease; however, reduced LAE and SAE [72] and impaired carotid and femoral arterial stiffness [76] was still seen in naïve participants even after adjusting for these risk factors in these studies. In addition, it was found that recently HIV-infected participants were not at increased risk of atherosclerotic disease when compared to uninfected controls [77], so well-treated HIV-seropositive participants, such as our ART treated group, may not vary significantly from healthy controls, as shown in our study.

Currently, the increased immune activation, increased expression of immune checkpoint inhibitors and chronic inflammation among HIV-infected individuals is thought to increase atherosclerotic progression and arterial stiffness [53, 54, 78]. We previously published that in ART naïve group, cardiac function was decreased with evidence of increased vascular resistance and that LAG3, PD1 or LAG3 plus PD1 expressing CD4 T cells were inversely correlated with cardiac function while being directly correlated with vascular resistance [78]. Our results show that both CD4 and CD8 T-cell activation was highest in treatment- naïve participants, and that this high immune activation correlated with lower cardiac function, such as lower stroke volume and cardiac output. Previous studies have shown that CD8 T-cell immune activation was more predictive of carotid plaque, while CD4 T-cell activation was associated with arterial stiffness [79, 80]. In our study, higher CD8 T-cell activation rather than CD4 T-cell activation was associated with markers of arterial stiffness, such as lower LAE and SAE. It has been demonstrated, however, that both activated CD4 + and CD8 + T cells can be associated with decreased arterial distensibility, likely due to both subtypes secreting pro-inflammatory mediators [80, 81]. Association of CD8 T cell immune activation with small and large artery elasticity in group 2 may be more associated with a role of the persistent immune activation on CVD outcomes over time. In group 1 ongoing viral replication and shorter duration of infection may not be able to capture the relationship of immune activation and arterial elasticity.

In ART-treated participants, the cardiac function was normalized, and no associations were seen with T-cell activation. This may be due to lower T-cell activation and improved cardiac functions seen in participants on ART.

The role of inflammatory mechanisms in the initiation and progression of CVD along with the rupture of atheromatous plaques has recently become more appreciated [55]. Various inflammatory markers, such as IL-6, TNFR-1, and TNFR-2, have been associated with CVD and increased mortality in HIV-positive participants [25, 27, 55–60]. In our study, IL-6 was associated with poor cardiac measures, including lower stroke volume and cardiac output, increased arterial stiffness, and higher SVR in naïve participants. Similarly, TNFR-1, TNFR-2, and to a lesser extent TNFα were also associated with markers of CVD. While still lower than naïve participants, immune activation of CD4 T-cells was higher in ART-treated compared to healthy participants, which could reflect ongoing immune activation even with successful suppression of HIV replication [25]. However, the risk of CVD is likely less severe given the lower immune activation of ART-treated participants compared to naïve. Inflammatory markers such as IL-6 not only correlated with CVD parameters, but also markers of microbial translocation, such as sCD14 [56].

Bacterial translocation has been implicated as a possible cause of immune activation leading to CVD progression [15, 34, 60]. In previous studies, elevated LPS and sCD14 were associated with increased coronary artery calcification and mortality in HIV infection, while in healthy individuals no association was found [21, 56, 59, 82, 83]. The naïve participants in our cohort had higher LPS and sCD14 markers, and both of these markers were associated with poor cardiovascular parameters, such as lower stroke volume and cardiac output, as well as increased arterial stiffness. LPS and sCD14 levels were lowest among participants that were ART-treated and healthy, likely due to the cardioprotective reduction in inflammatory markers and improved immune function [71, 84, 85].

## Conclusions

Although it was previously thought that virologic suppression came at the expense of possible pro-atherogenic side effects of ART, our data suggest that the overall benefits of treatment in terms of virologic suppression with concomitant reduction in inflammatory markers and improvement of cardiac function are likely to be cardioprotective, ART treated patients had improved cardiac function, with Group 2 being comparable to healthy participants. Our data does not indicate how long they have to be on ART to start manifesting improvement in cardiac measures as the median duration of their treatment was 43 months (range 22–72 months). As ART regimens have improved dramatically and continue to improve in rapidity of achieving virus suppression, this is a moving target. Although the data were adjusted for all of the known risk factors, lack of information about other potential confounders such as other co-infections, like CMV may present among our study populations, and that could be a limitation with any cross-sectional study. Longitudinal studies are necessary to further explore and confirm the deterioration or improvement of arterial stiffness in participants on ART. Taken together, our data points towards the detrimental effects of persistent immune activation in CD4 T-cell populations on cardiac outcomes. Early ART initiation irrespective of CD4 counts may prevent adverse CVD outcomes by lowering immune activation, and thus limiting complications and morbidity in HIV-infected individuals.

## Supplementary Information

**Additional file 1. Table S1**. Measures of cardiac functioning, and arterial stiffness in naïve and ART-treated participants stratified by nadir CD4 counts.** Table S2**. Comparison of T-cell activation, plasma inflammatory markers, and MT markers in HIV-infected naïve participants, on ART participants, and HIV-uninfected control groups.

## Data Availability

The datasets used and/or analyzed during the current study are available from the corresponding author on request.

## Abbreviations

PWH: People with HIV
MT: Microbial translocation
ART: Antiretroviral therapy
C-IMT: Carotid intima-media thickness
IA: Immune activation
CVD: Cardiovascular disease
SMART: Strategies for management of antiretroviral therapy
VAC: Veterans aging cohort
TNFR: Tumor necrosis factor receptor
sCD14: Soluble CD14
LPS: Lipopolysaccharide
PWV: Pulse-wave velocity
INSIGHT: International network for strategic initiatives in Global HIV trials
START: Strategic timing of anti-retroviral treatment
LAE: Large artery elasticity index
SAE: Small artery elasticity index
SVR: Systemic vascular resistance
TVI: Total vascular impedance
