# Supplementary material for: Immune correlates of cardiovascular co-morbidity in HIV infected participants from South India
Source: BMC Immunol. 2022 May 17;23:24. doi: 10.1186/s12865-022-00498-0 (PMC9115939; doi:10.1186/s12865-022-00498-0)
Supplement: Supplementary file 1 — Additional file 1. Table S1. Measures of cardiac functioning, and arterial stiffness in naïve and ART-treated participants stratified by nadir CD4 counts. Table S2. Comparison of T-cell activation, plasma inflammatory markers, and MT markers in HIV-infected naïve participants, on ART participants, and HIV-uninfected control groups. [file 12865_2022_498_MOESM1_ESM.docx]

**Supplement Information**

**Table S1:Measures of cardiac functioning, and arterial stiffness in naïve and ART-treated participants stratified by nadir CD4 counts:**

| **Clinical parameters** | **Group 1a; (n=29)** | **Group 1b; (n=22)** | **Group 1c; (n=51)** | ***P*; Group 1a vs 1b** | ***P*; Group 1a vs 1c** | ***P*; Group 1b vs 1c** | **Group 2a; (n=51)** | **Group 2b; (n=51)** | **Group 2c; (n=70)** | ***P*; Group 2a vs 2b** | ***P*; Group 2a vs 2c** | ***P*; Group 2b vs 2c** |
| --- | --- | --- | --- | --- | --- | --- | --- | --- | --- | --- | --- | --- |
| Estimated cardiac ejection time (msec) | 263.17±35.8 | 287.41±24.84 | 294.88±31.62 | 0.006* | <0.001* | 0.329 | 298.08±36.66 | 301.96±35.23 | 304.83±31.48 | 0.571 | 0.292 | 0.625 |
| Estimated stroke volume (ml/beat) | 46.17±19.41 | 63.40±14.89 | 64.78±15.16 | 0.001* | <0.001* | 0.722 | 67.84±17.73 | 70.05±16.24 | 68.6±15.46 | 0.512 | 0.803 | 0.617 |
| Estimated stroke volume index (ml/beat/m^2^) | 29.89±11.04 | 39.09±8.34 | 39.82±8.34 | 0.001* | <0.001* | 0.732 | 40.47±10.46 | 42.03±9.22 | 42.58±8.23 | 0.424 | 0.216 | 0.732 |
| Estimated cardiac output (L/min) | 3.93±1.09 | 4.8±0.727 | 5.05±0.842 | 0.002* | <0.001* | 0.229 | 5.16±0.927 | 5.09±0.719 | 4.93±.0.812 | 0.66 | 0.201 | 0.357 |
| Estimated cardiac index (L/min/m^2^) | 2.55±0.605 | 2.96±0.334 | 3.10±0.44 | 0.004* | <0.001* | 0.179 | 3.03±0.435 | 3.04±0.323 | 3.08±0.354 | 0.837 | 0.516 | 0.622 |
| Large artery elasticity index (ml/mmHg x 10) | 10.58±5.15 | 13.91±4.63 | 13.45±3.87 | 0.021* | 0.006* | 0.661 | 14.41±4.67 | 15.40±5.17 | 14.53±4.51 | 0.31 | 0.885 | 0.325 |
| Small artery elasticity index (ml/mmHg x 100) | 3.90±1.72 | 4.4±1.98 | 4.47±1.96 | 0.344 | 0.061 | 0.507 | 5.01±2.56 | 5.48±2.22 | 5.09±2.44 | 0.326 | 0.868 | 0.366 |
| Systemic vascular resistance (dyne.sec.cm^-5^) | 1888.6±686.66 | 1434.5±293.74 | 1453.9±493.49 | 0.003* | 0.004* | 0.865 | 1492.9±748.77 | 1343.1±306.10 | 1395.3±263.61 | 0.189 | 0.376 | 0.317 |
| Right IMT (in cm) | 0.0525±0.0058 | 0.0545±0.0105 | 0.0535±0.014 | 0.421 | 0.654 | 0.767 | 0.0522±0.0118 | 0.05±0.0116 | 0.0522±0.0118 | 0.349 | 0.956 | 0.274 |
| Left IMT (in cm) | 0.0529±0.0101 | 0.0564±0.01329 | 0.0483±0.0105 | 0.295 | 0.072 | 0.008* | 0.0528±0.0129 | 0.0522±0.0113 | 0.0528±0.0158 | 0.791 | 0.438 | 0.291 |

Group 1a; Naïve patients with nadir CD4 T-cell count <200 cells/µL, Group 1b; Naïve patients with nadir CD4 T-cell count 200-350 cells/µL, Group 1c; Naïve patients with nadir CD4 T-cell count >350 cells/µL, Group 2a; On ART patients with nadir CD4 T-cell count <200 cells/µL, Group 2b; On ART patients with nadir CD4 T-cell count 200-350 cells/µL, Group 2c; On ART patients with nadir CD4 T-cell count >350 cells/µL. All continuous variables are presented as mean ± standard deviation. Mann-Whitney U-test was used to calculate *p*-values between the study groups. * Indicates statistical significance

**Table S2: Comparison of T-cell activation, plasma inflammatory markers, and MT markers in HIV-infected naïve participants, on ART participants, and HIV-uninfected control groups:**

| **Parameter** | **HIV+ Group 1; Naïve participants (n=102)** | **HIV+ Group 2; Participants on ART (n=172)** | **Group 3; HIV-uninfected controls (n=64)** | ***P-*value; Group 1 vs 2** | ***P-* value; Group 1 vs 3** | ***P-* value; Group 2 vs 3** |
| --- | --- | --- | --- | --- | --- | --- |
| CD4 +HLA-DR^+^CD38^+^) | 8.24 (6.23, 14) | 3.4 (2.63, 4.5) | 2.8 (2.12, 3.45) | <0.001* | <0.001* | <0.001* |
| CD8+HLA-DR^+^CD38^+^) | 46.1 (31.3, 56.3) | 15.2 (9.82, 21.6) | 14.8 (10.5, 23.4) | <0.001* | <0.001* | 0.676 |
| IFNα2 (pg/mL) | 2.2 (1.94, 2.2) | 1.94 (0.24, 1.98) | 1.98 (1.94, 1.98) | <0.001* | 0.011* | 0.266 |
| IFNg (pg/mL) | 0.96 (0.23, 3.28) | 0.96 (0.14, 1.74) | 0.96 (0.14, 2.64) | 0.044* | 0.353 | 0.464 |
| IL10 (pg/mL) | 0.34 (0.014, 3.63) | 0.021 (0.014, 0.34) | 0.021 (0.014, 0.34) | <0.001* | 0.017* | 0.113 |
| IL12 (pg/mL) | 0.28 (0.15, 0.28) | 0.18 (0.15, 0.33) | 0.28 (0.18, 0.33) | 0.417 | 0.145 | 0.043* |
| IL17 (pg/mL) | 0.091 (0.06, 0.33) | 0.12 (0.02, 0.61) | 0.12 (0.06, 0.51) | 0.957 | 0.512 | 0.539 |
| IL1b (pg/mL) | 0.11 (0.01, 0.11) | 0.0615 (0.01, 0.28) | 0.113 (0.007, 0.28) | 0.852 | 0.968 | 0.222 |
| IL2 (pg/mL) | 0.19 (0.19, 0.22) | 0.19 (0.19, 0.22) | 0.19 (0.14, 0.22) | 0.487 | 0.286 | 0.698 |
| IL6 (pg/mL) | 0.15 (0.06, 1.42) | 0.021 (0.014, 0.15) | 0.021 (0.014, 0.105) | <0.001* | <0.001* | 0.799 |
| TNF-α (pg/mL) | 14.46 (9.36, 24.34) | 7.58 (5.67, 10.53) | 5.93 (4.08, 8.24) | <0.001* | <0.001* | 0.001* |
| TNFR-1 (pg/mL) | 2552.4 (1735.8, 3367.1) | 2160.2 (1809.7, 2723) | 2273.8 (1880.9, 2568.6) | 0.01* | 0.036* | 0.914 |
| TNFR-2 (pg/mL) | 755.36 (588.48, 968.94) | 536.64 (421.22, 707.5) | 512.7 (397.5, 665.24) | <0.001* | <0.001* | 0.608 |
| sCD14 (ng/mL) | 2791.3 (2044.7, 3908.3) | 3098.9 (2371.9, 3836.8) | 2485.2 (2036.4, 2835.5) | 0.147 | 0.004* | <0.001* |
| LPS (pg/mL) | 193 (165, 234) | 164 (143, 196.5) | 163 (138.5, 190) | <0.001* | <0.001* | 0.611 |

All continuous variables are presented as median and interquartile ranges. Mann-Whitney U-test was used to calculate *p*-value between the study groups. * Indicates statistical significance.
